# The LINC00261/MiR105-5p/SELL axis is involved in dysfunction of B cell and is associated with overall survival in hepatocellular carcinoma

**DOI:** 10.7717/peerj.12588

**Published:** 2022-06-09

**Authors:** Hao Song, Xing-Feng Huang, Shu-yang Hu, Lei-Lei Lu, Xiao-Yu Yang

**Affiliations:** 1 Department of Organ Transplantation, Shanghai Eastern Hepatobiliary Surgery Hospital, Shanghai, China; 2 Origimed Co. Ltd, Shanghai, China

**Keywords:** Immune cell landscape, SELL, LINC00261, miR105-5p, B cell, Hepatocellular carcinoma

## Abstract

**Background:**

Previous studies have been reported the immune dysfunction of various live tissues. However, the potential molecular mechanism of post-transcriptional regulation of immune related genes in hepatocellular carcinoma (HCC) is still not clear. We tried to identify crucial immune related biomarkers associated with HCC patients’ outcomes and to reveal the transcriptional regulation.

**Method:**

The fractions of 22 immune cells in tumor and adjacent tissues were estimated by CIBERSORT. Kruskal-Wallis test and differentially expressed analyzes were used for comparative studies. Cox proportional hazard regression model, Kaplan-Meier estimates and Log-rank test were used for survival analyses.

**Results:**

From The Cancer Genome Atlas (TCGA), the gene, lncRNA and miRNA expression profiles of 379 HCC samples with clinical information were used for comparative studies. Eleven adaptive and innate immune cell types were significantly altered in HCC samples, including B cell memory, regulatory T cells and follicular helper T cells. Differentially expressed competing endogenous RNA (ceRNA) network associated with patients’ overall survival was identified. Then, the novel pathway, including LINC00261, MiR105-5p and selectin L(SELL) was found and may be potential novel biomarkers for patients’ outcomes and immunotherapy. Furthermore, SELL was significantly positively correlated (correlation coefficients: 0.47–0.69) with 12 known gene signatures of immunotherapy except for programmed cell death 1 (PDCD1).

**Conclusions:**

Our findings could provide insights into the selection of novel LINC00261/MiR105-5p/SELL pathway which is associated with overall survival and may impact on efficacy of immunotherapy in HCC.

## Introduction

Liver cancer is one of the most common malignancies with a high mortality rate worldwide. Approximately 75–85% of primary liver cancer cases are hepatocellular carcinoma (Bray et al., 2018). Previous studies have identified many of the main risk factors for HCC, including: hepatitis B virus (HBV) infection, hepatitis C virus (HCV) infection, aflatoxin exposure, smoking, alcohol, and obesity (Marengo, Rosso & Bugianesi, 2016). Although lots of the molecular mechanisms of HCC have been uncovered, the mechanisms associated with an early-stage diagnosis of HCC is still unknown. For HCC patients in the advanced stage, only a few drugs, such as sorafenib, regorafenib and nivolumab, have been approved (Bruix et al., 2017; El-Khoueiry et al., 2017). Recently, many studies have reported that non-coding RNAs (ncRNAs) play key roles in HCC and may also be novel biomarkers and therapeutic targets for the disease (Chi et al., 2019).

It is well known that approximately 70–80% of the human genome is not protein-encoding and is transcribed as various ncRNAs including long non-coding RNA (lncRNA), microRNA (miRNA) (Lanzafame et al., 2018), tRNA (Yu et al., 2020), rRNA (Kaliatsi et al., 2020) and snoRNAs (Xing & Chen, 2018). Among these, there has been a focus on the lncRNAs and miRNAs as important regulators of post-transcriptional gene expression. The lncRNA is defined as ncRNA greater than ~200 nucleotides and the miRNA is defined as small ncRNAs of approximately 20–24 nucleotides in length (Zhang, Zhang & Zhang, 2016). MiRNAs have recently been shown to inhibit gene expression through the post-transcriptional regulation of mRNA. Meanwhile, some competitive endogenous RNA (ceRNA) can indirectly regulate the expression of target genes through competitively binding the sites which can also be targeted by miRNAs. Many lncRNAs can also act as ceRNAs to influence the expression levels of specific miRNA target genes (Tam et al., 2019).

Both lncRNAs and miRNAs play important roles in the regulation of biological processes, including cell growth, proliferation, migration and apoptosis in HCC (Tam et al., 2019). The HUCL lncRNA can reduce the expression and activity level of MiR372 in liver cancer cells (Wang et al., 2010). HULC can also increase the expression of E2F1 (E2F transcription factor 1) by competing with MiR107, which targets E2F and accelerates EMT (epithelial-mesenchymal transformation) through MiR200a-3p/ZEB1 (zinc finger E-box binding homeobox 1) signaling (Li et al., 2016; Lu et al., 2016). The HOTAIR lncRNA can promote the EMT of HCC cells by regulating ZEB1 through sponging miR-23b-3p (Yang et al., 2018b). The NEAT1 lncRNA (nuclear paraspeckle assembly transcript 1) has been reported to be a regulator of the STAT3 (signal transducer and activator of transcription 3) pathway promoting cell growth through MiR124-3p (Liu et al., 2018) and MiR384 (Zhu et al., 2018). In HepG2 cells, increased SNHG1 (small nucleolar RNA host gene 1) can reduce the expression level of MiR195 to promote HCC cell proliferation, invasion, and migration (Zheng et al., 2018). Although there have been an increasing number of studies on ncRNAs, the molecular mechanism of ncRNAs in HCC development, metastasis, and recurrence remains largely unknown (Lanzafame et al., 2018).

In recent years, the tumor microenvironment (TME) has been considered a target-rich environment for anticancer agents (Hanahan & Coussens, 2012). The CIBERSORT tool was developed to quantify 22 immune cell types (Newman et al., 2015). It has evaluated the roles of innate and adaptive immune dysfunction which can alter tumor activities and affect clinical outcomes in HCC with different degrees of fibrosis (Tang et al., 2019). Meanwhile, ncRNAs also serve as the key regulators in both innate immunity and adaptive immunity (Carpenter et al., 2013), but the mechanisms through which ncRNAs regulate immune responses are still unclear. In this study, we used CIBERSORT to estimate the proportions of different immune cells in HCC samples and then sought to identify the molecular mechanisms of lncRNAs, miRNAs and their targets of immune dysfunction. Our findings provide new insights into the application of immunotherapies in HCC. (Wu et al., 2021).

## Materials and Methods

### Expression profiles

The gene, lncRNA and miRNA expression profiles from 379 samples (337 HCC tumors and 42 adjacent tissues, Table S1) were downloaded from the UCSC Xena platform (https://xenabrowser.net/datapages/, the dataset named “GDC TCGA Liver Cancer (LIHC) (14 datasets)”) which has stored all the TCGA public datasets of various cancers (Goldman et al., 2020). These samples had detailed clinical outcomes including overall survival (OS). The independent microarray datasets of GSE115019 (Shi et al., 2018) and GSE14520 (Sun et al., 2017) were downloaded from the gene expression omnibus (https://www.ncbi.nlm.nih.gov/geo/) and used for validation.

### Immune cell landscape by CIBERSORT

CIBERSORT is an analytical tool which accurately quantifies the relative levels of distinct immune cell types within a complex gene expression mixture (Newman et al., 2015). To quantify the fractions of 22 immune cells, CIBERSORT was used for all HCC samples based on 547 genes.

### Differentially expressed genes/lncRNAs/miRNAs and the ceRNA network

Differentially expressed genes (DEGs) (or lncRNA (DELs) and miRNAs (DEMs)) were identified between different liver tissues using R package ‘limma.’ For DEGs, the threshold of absolute fold-change was more than 2. For DELs and DEMs, the threshold of absolute fold-change was more than 1.5 (Li et al., 2021; Weiner et al., 2021). For DEGs/DELs/DEMs, the adjusted p-value was less than 0.05. Stem loop miRNAs identified in TCGA were then mapped into mature miRNAs using miRBase (V22.1) (Kozomara, Birgaoanu & Griffiths-Jones, 2019) and then the MirTarget algorithm was used to predict the regulation between mature miRNAs and target genes with the prediction score >80 (Liu & Wang, 2019). The candidate miRNA-gene pairs were validated by TargetscanHuman (Version 7.2) (Agarwal et al., 2015). Meanwhile, the interactions of lncRNAs and miRNAs were predicted using Starbase (Version 2) (Li et al., 2014). A ceRNA regulation network of all interactions was visualized using Cytoscape (Version 3.7.1) (Shannon et al., 2003). For validation, the differentially expressed genes/lncRNAs/miRNAs were also identified by GSE115019.

### Statistical analysis

The GO and KEGG enrichment analyses were calculated using the R package ‘clusterprofiler.’ The DEGs (absolute fold-change > 2) were used for the enrichment analysis and all significant results were selected with a *P*-value < 0.05. A COXPH (COX Proportional Hazards) regression analysis was carried out to assess whether the expression levels of the candidate genes/lncRNAs/miRNAs were significantly associated with the overall survival (OS) of HCC patients. For the survival analysis, the HCC patients were divided into high and low categories based on the mean expression levels of the candidate biomarkers. Then, for validation, a Kaplan-Meier survival analysis and Log-rank test were carried out in both the TCGA data set and the independent gene expression profile (GSE14520). To analyze the possible correlations among SELL and other well-known immune-related genes (Socinski et al., 2018), a Pearson correlation analysis was performed and the correlation coefficients were used for further studies. All of the statistical analyses were calculated using R language (Version 3.5.3).

## Results

### Patient characteristics and processing

A total of 379 samples (337 HCC tumors and 42 adjacent tissues) were selected from the UCSC Xena platform (Goldman et al., 2020) with the accompanying gene, lncRNA and microRNA profiles (Table S1). Among the 337 HCC patients, 231 were male and 106 were female. The median patient age was 61 years old (range:16–85) and the majority of the patients were white (160, 47.5%) or Asian (153, 45.4%). There were 254 patients in the early (stage I and II) TNM (Tumor, Node, Metastasis) stages and 83 in the advanced (stage III and IV) stages. The Child-Pugh score was recorded for 226 of the patients with the overwhelming majority (90.7%, 205/226) having a score in class A.

**Figure 1:**
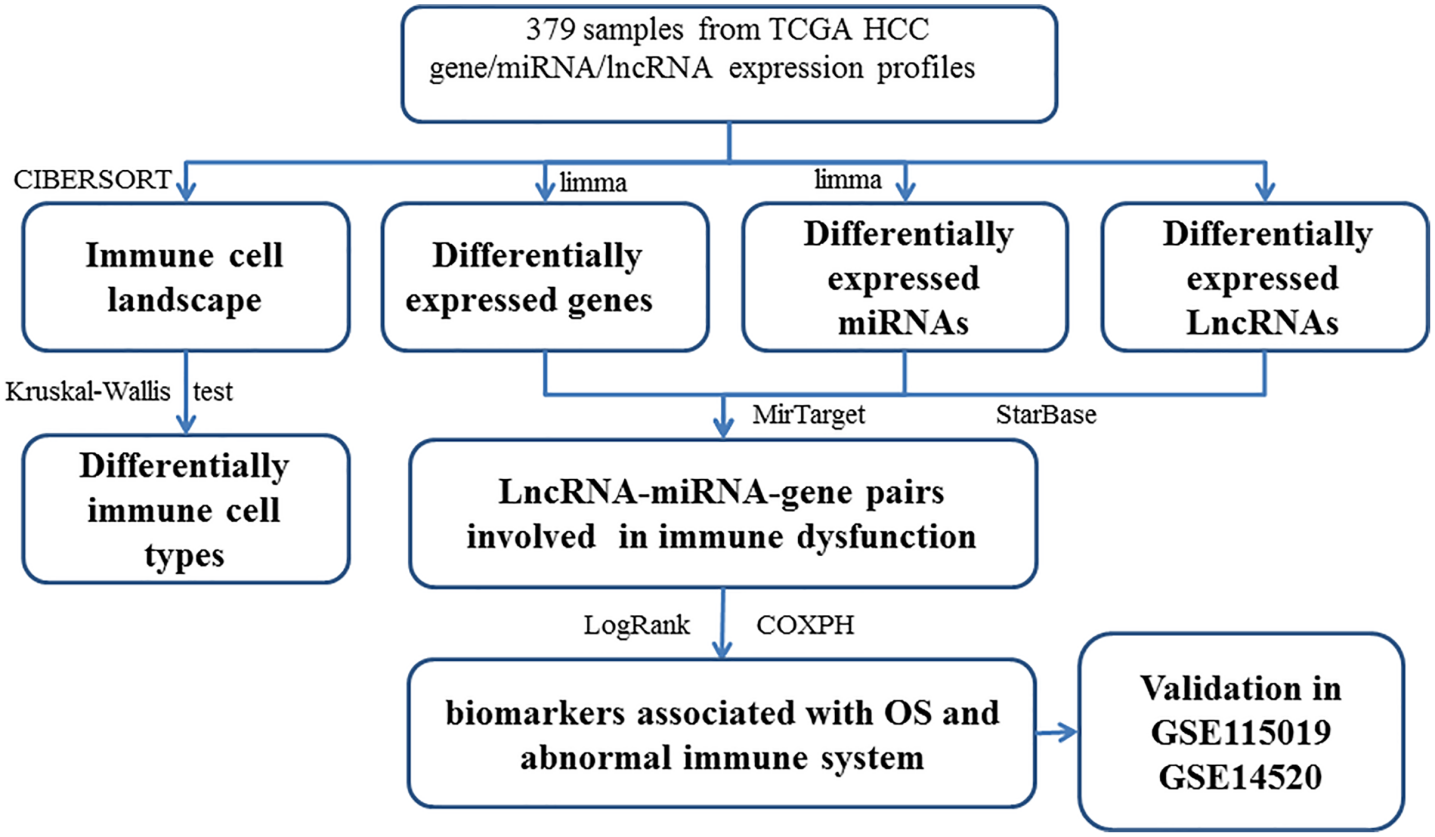
The workflow of integrative analysis of ceRNA network regulated immune system in HCC.

The DEGs (or DELs and DEMs) were identified between different tissues in order to reveal the molecular mechanisms. Then, 3705 DEGs (1,628 up-regulated and 2,077 down-regulated), 216 DELs (179 up-regulated and 37 down-regulated), and 201 DEMs (87 up-regulated and 114 down-regulated) were found in tumor tissues. The study workflow is shown in Fig. 1. The top 10% up-regulated and down-regulated DEGs are shown in Table S6. For the DEGs, the enrichment results of the top 20 Gene Ontology BPs (biological process) are shown in Table S2. The increased DEGs had significant dysfunctions in cell division and DNA replication while the decreased DEGs had significant dysfunctions in cell adhesion, inflammatory responses and immune responses. The enrichment results of the top 20 KEGG pathways are shown in Table S2, including the metabolic pathway, cell cycle, DNA replication, focal adhesion, ECM-receptor interaction and signaling pathways (p53, PI3K (phosphatidylinositol 3′ -kinase)-Akt, PPAR (Peroxisome proliferator-activated receptor)) which had been reported to be associated with HCC.

### Different proportions of immune cells types in HCC

Our study focused on disordered, innate and adaptive immunity. First, the proportions of 22 immune cell types of the 379 HCC samples were estimated based on the LM22 signature files using CIBERSORT (results shown in Table S3). Then, the comparative studies were carried out with the results showing that fractions of 11 immune cell types were significantly different between the tumor and adjacent tissues.

**Figure 2:**
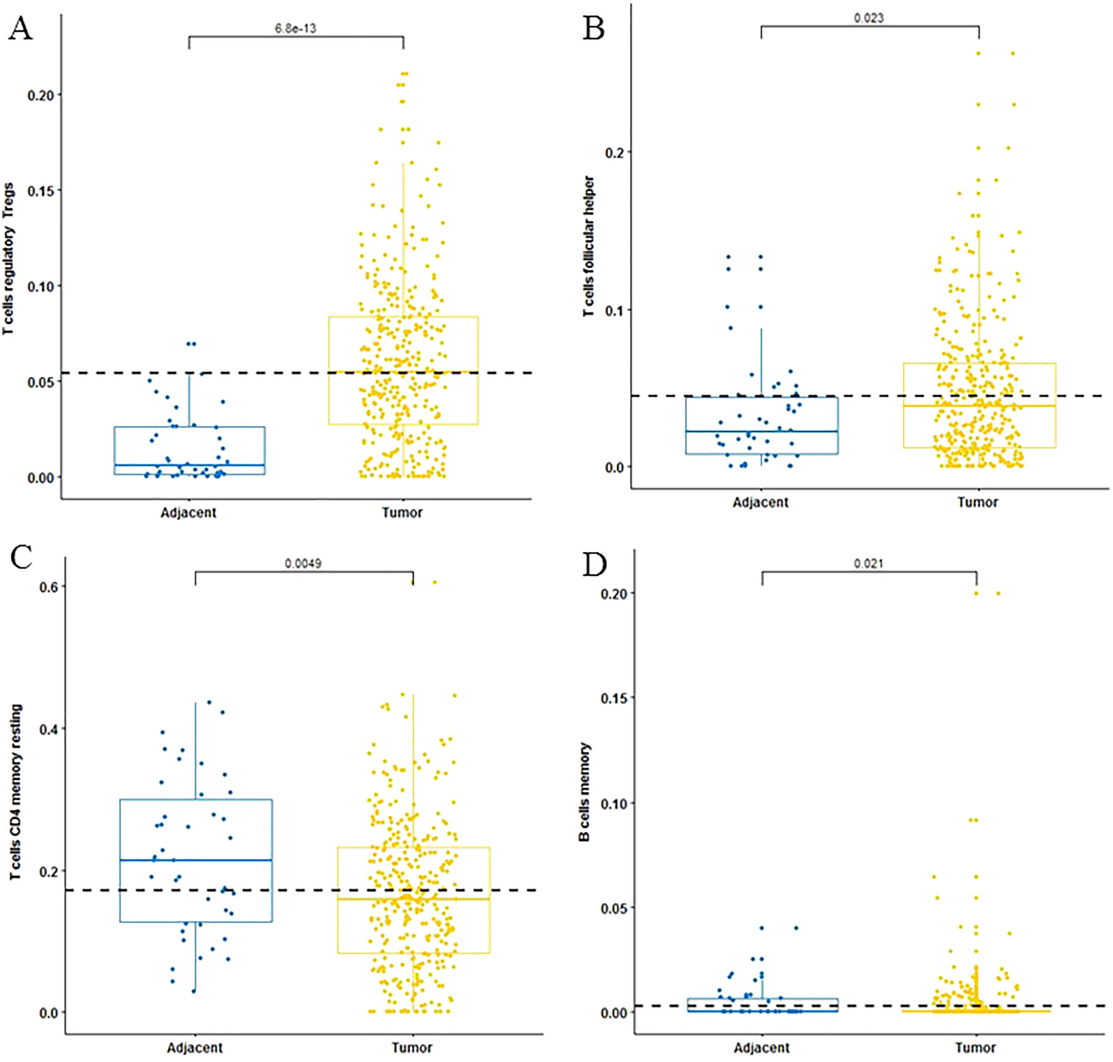
The fractions of adaptive immune cells in TCGA dataset. CIBERSORT immune cell fractions were determined for each sample; each dot represents one sample. Mean values and standard deviations for each cell subset including the regulatory Tregs (A), follicular helper (B), CD4 memory resting (C) and B cell memory (D) were calculated for each sample group and compared using the Kruskal-Wallis test and the Wilcoxon test.

**Figure 3:**
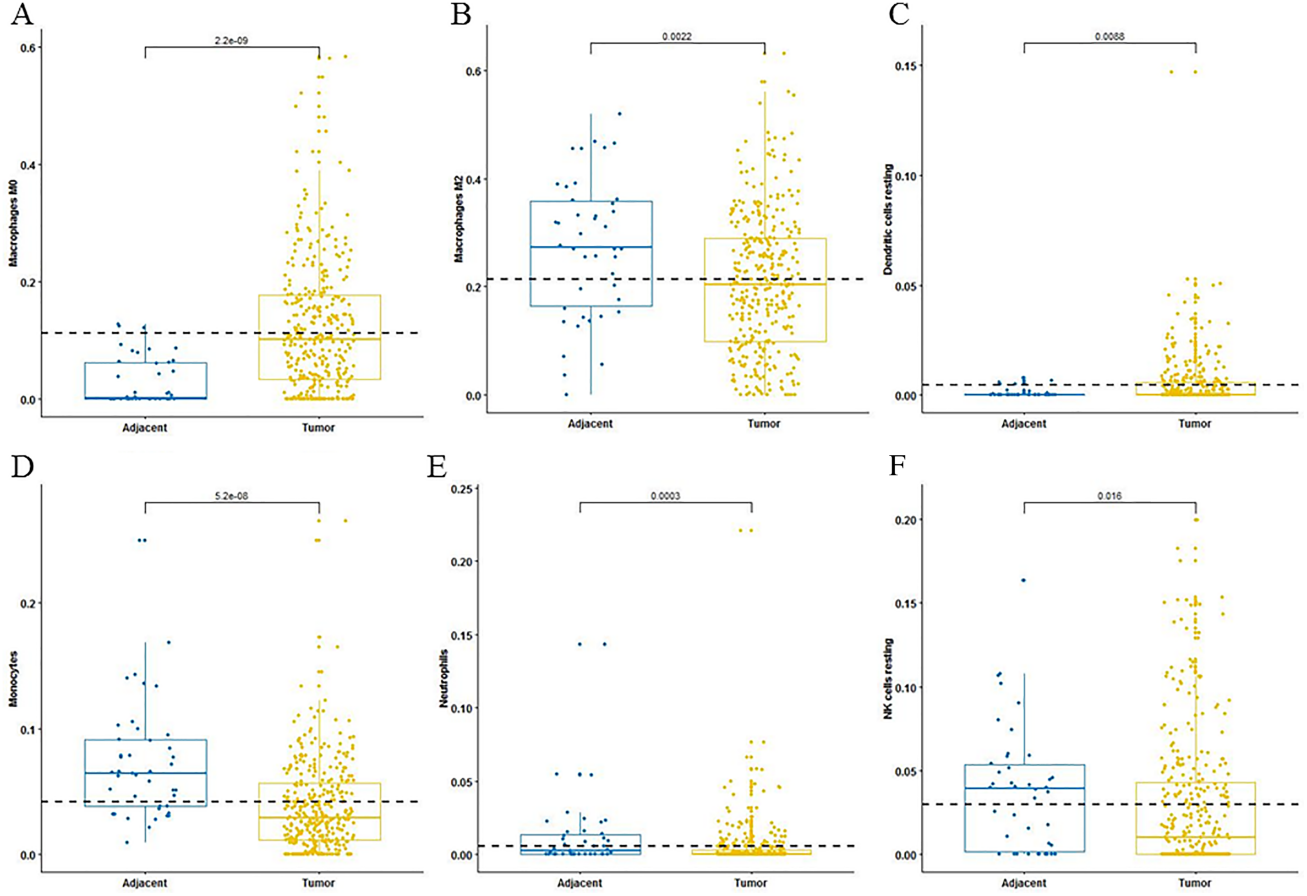
The fractions of innate immune cells in TCGA dataset. CIBERSORT immune cell fractions were determined for each sample; each dot represents one sample. Mean values and standard deviations for each cell subset including macrophages M0 (A), macrophages M2 (B), resting dendritic cells (C), neutrophils (D), monocytes (E) and resting NK cells (F) were calculated for each sample group and compared using the Kruskal-Wallis test and the Wilcoxon test.

For adaptive immune cells, three main T cell subpopulations were significantly different. In the tumor tissues, the regulatory T cells (Fig. 2A) and T follicular helper cells (Fig. 2B) were significantly increased compared to the adjacent tissues. Conversely, there were less resting memory CD4 cells in the tumor tissues (Fig. 2C). The fractions of memory B cells (Fig. 2D) and plasma cells in the tumors were significantly higher than adjacent tissue. For innate immune cells, six immune cell types were significantly altered between the two different tissues. There were three types of macrophages studied: M0 macrophages were increased in tumor samples, (Fig. 3A) M2 macrophages were decreased (Fig. 3B), and there was no difference in M1 macrophages between the two tissues. Four other types of immune cells, including resting dendritic cells, neutrophils, monocytes and resting NK cells were significantly higher in adjacent tissues (Figs. 3C–3F, respectively). Meanwhile, both of the resting and activated mast cells were not significantly altered.

### The differentially expressed ceRNA network associated with OS

**Figure 4:**
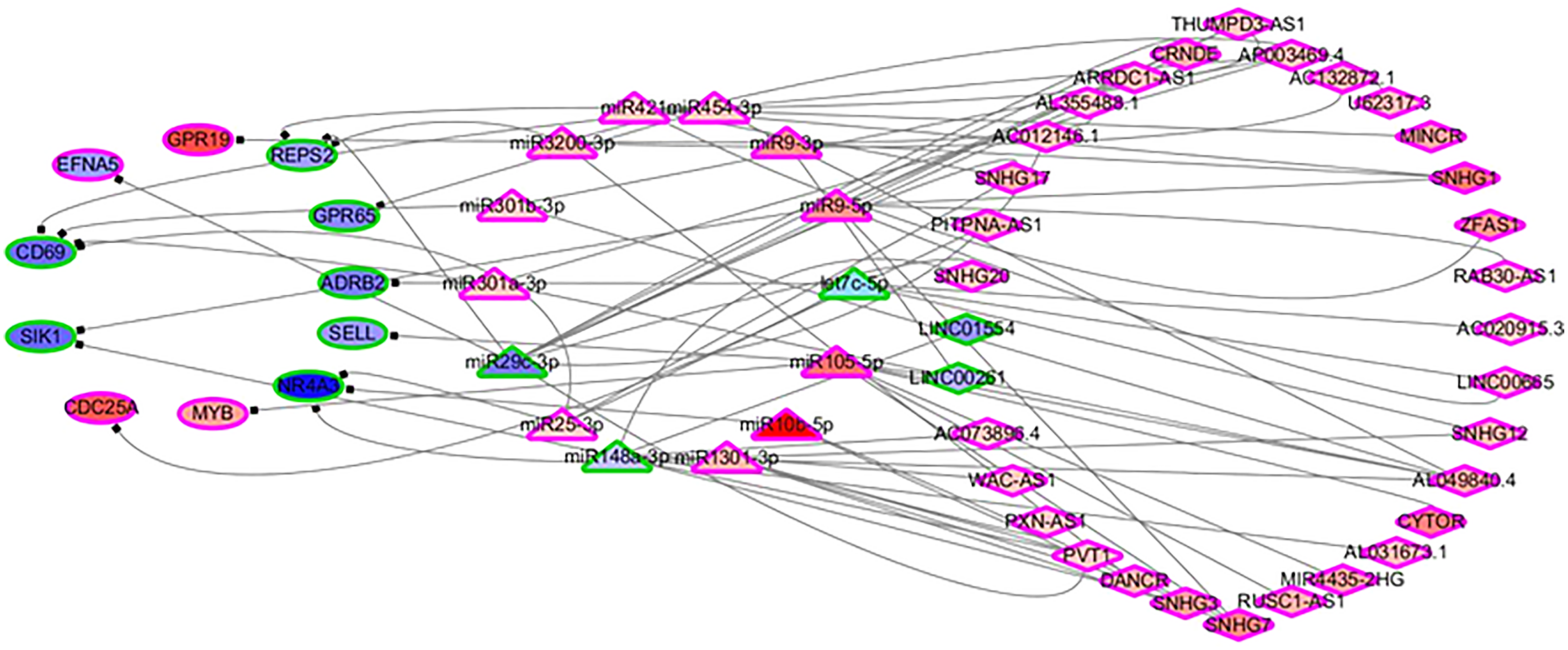
The differentially expressed ceRNA network associated with OS. Ellipses represent immune genes targeted by miRNAs, triangles represent miRNAs and diamonds represent lncRNAs. Red fill color indicates upregulated and blue one indicates downregulated. Red border indicates HR > 1 and green border indicates HR < 1. The regulatory relationships between miRNA and genes were from MirTarget, and the interactions between miRNA and lncRNAs were from StarBase.

In order to identify the mechanisms of the various immune cells, the differentially expressed ceRNA network, including three types of biomarkers (DEGs, DELs and DEMs) and their interactions, were predicted using miRbase (Kozomara, Birgaoanu & Griffiths-Jones, 2019), MirTarget (Liu & Wang, 2019) and Starbase (Li et al., 2014). Previous studies have found that the alterations of immune cells play critical roles in patient outcomes (overall survival and/or recurrence-free survival), so a COXPH analysis was carried out for further selection. Finally, 57 biomarkers (11 DEGs, 32 DELs and 14 mature DEMs) and 76 interactions (19 miRNA-gene pairs and 57 lincRNA-miRNA pairs) were selected. The differentially expressed ceRNA network is visualized in Fig. 4 (Tables S4, S5).

**Figure 5:**
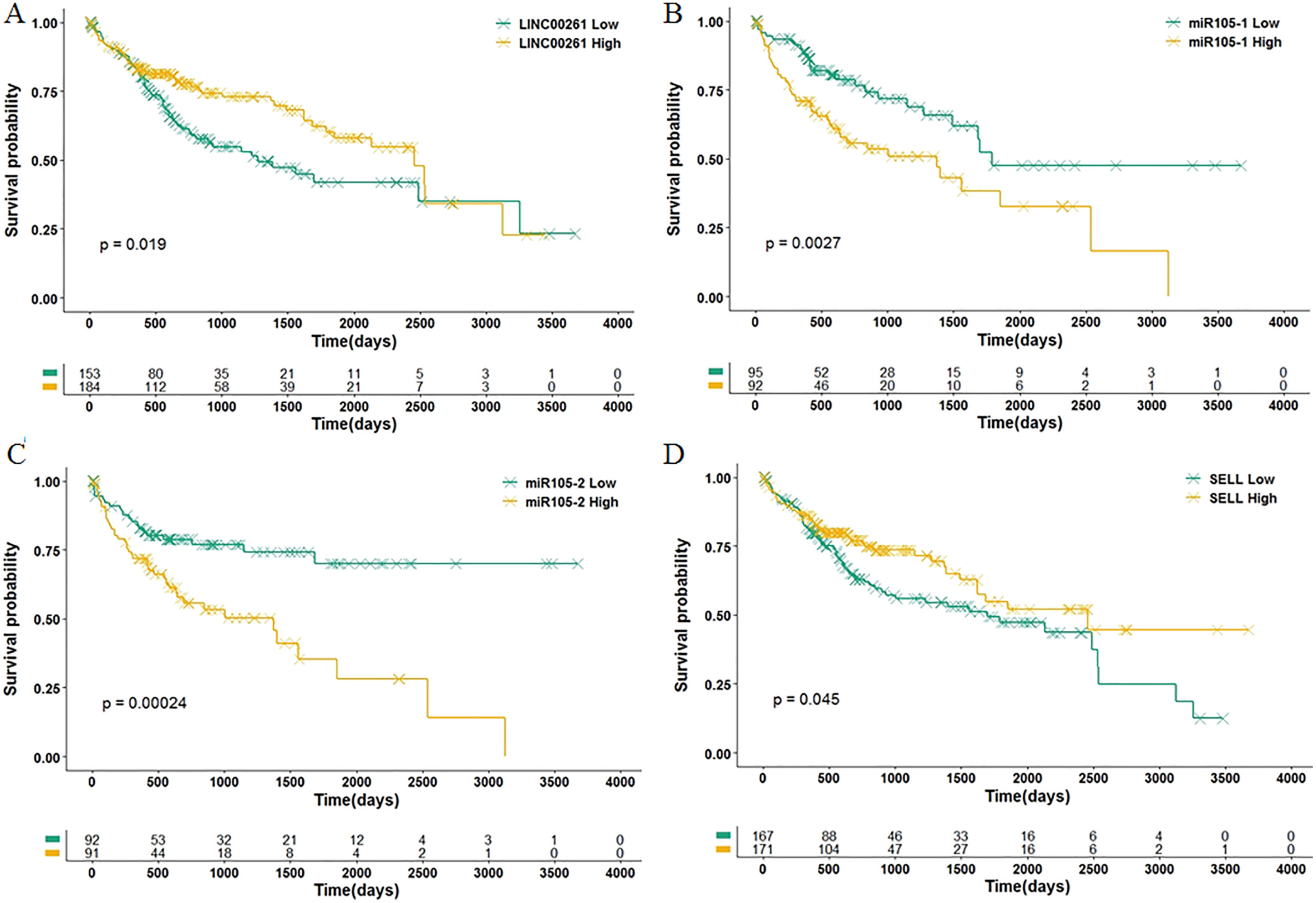
Association of biomarkers with OS. Patients were stratified into high and low categories based on the expression level of LINC00261 (cutoff = 4.26), MiR105-1 (cutoff = 1.64), MiR105-2 (cutoff = 1.65) and SELL (cutoff = 6.38) for Kaplan-Meier survival analysis by OS, and the results are shown in (A–D), respectively.

Among 11 DEGs, three genes involved with B cells—SELL (selectin L), SIK1 (salt inducible kinase 1) and CD69 (CD69 molecule)—were significantly decreased in HCC patients and significantly associated with OS. Further studies have identified a subnet that includes SELL, MiR105-5p (directly targeting SELL) and LINC00261 (directly interacting with MiR105-5p). The expression levels of SELL (Log2-fold-change = −1.14) and LINC00261 (Log2-fold-change = −0.90) were significantly decreased in tumor tissues compared to the adjacent tissues. In contrast, stem loop sequence MiR105-5p, comprised of MiR105-1 and MiR105-2, was significantly increased in HCC samples. Meanwhile, the results of the COXPH analyses, Kaplan-Meier analyses and Log-rank test suggested that MiR105-1 (*P* = 6.27E−5, HR = 1.14(1.07–1.22), Fig. 5B) and MiR105-2 (*P* = 8.08E−5, HR = 1.14(1.07–1.21), Fig. 5C) were negatively correlated to OS, whereas SELL (*P* = 8.10E−2, HR = 0.836(0.732–0.954), Fig. 5D) and LINC00261 (*P* = 3.84E−2, HR = 0.848(0.726–0.991), Fig. 5A) were positively associated with OS.

### Validation and potential molecular mechanisms

**Figure 6:**
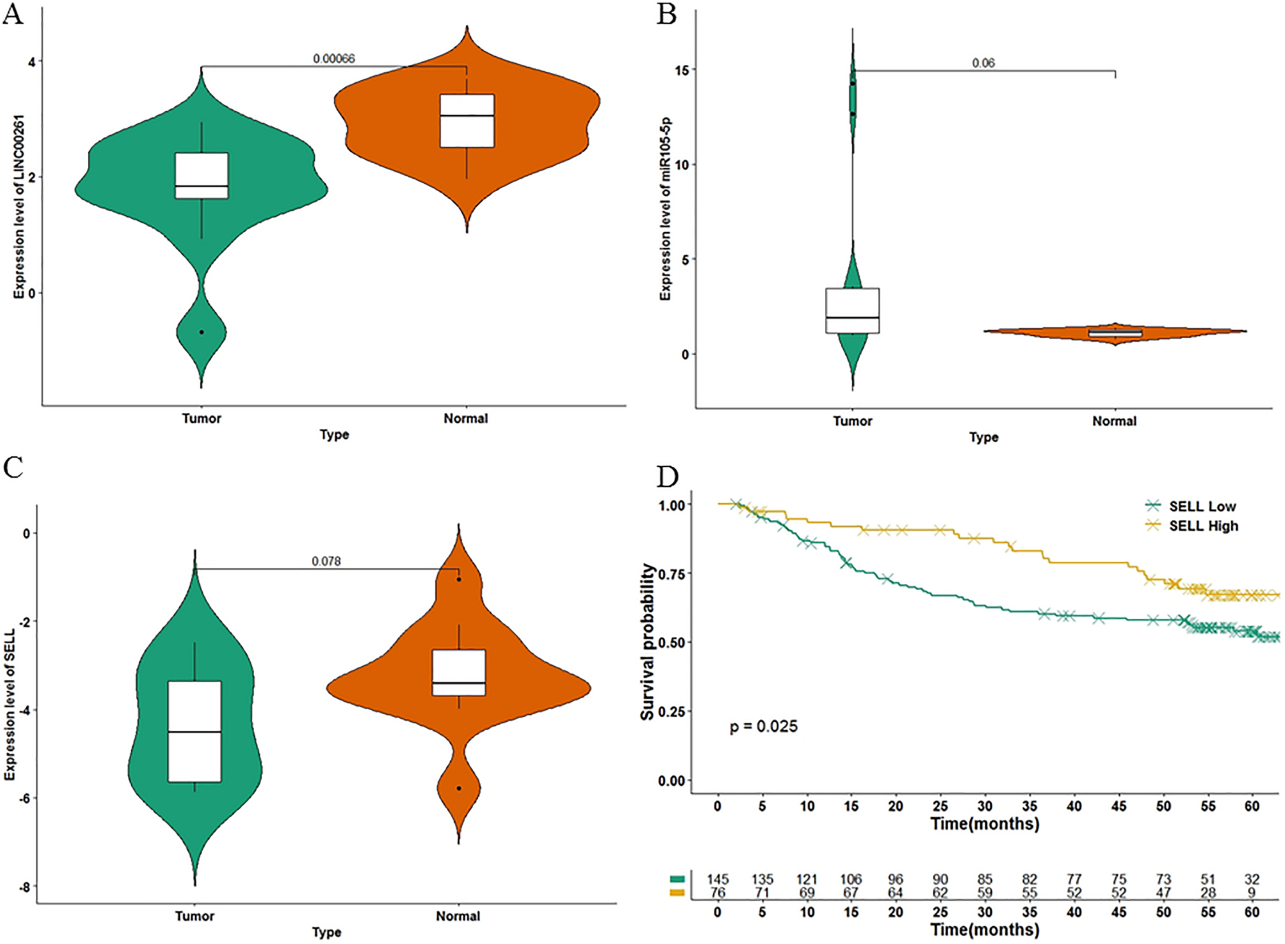
The validation in two independent datasets. In GSE115019, the expression levels of LINC00261 (A), MiR105-5p (B) and SELL (C) were calculated. In GSE14520, 221 HCC samples were stratified into high and low categories based on the expression level of SELL for Kaplan-Meier survival analysis by OS (D).

**Figure 7:**
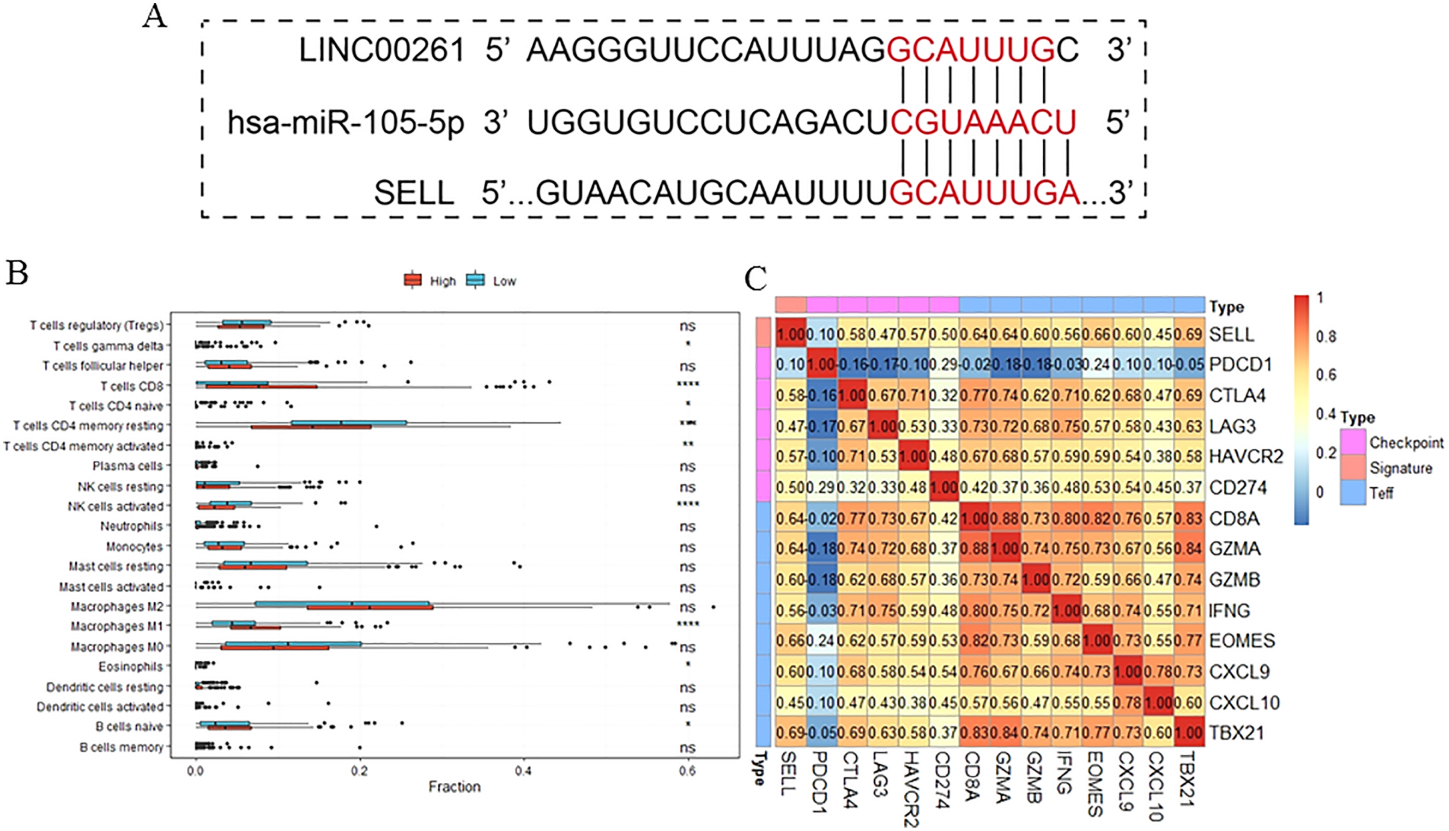
The function of SELL and validation of the regulation. Patients were divided into two groups according to expression level of SELL. The regulation was predicted by TargetScanHuman (A). The different fractions of immune cell subtypes were then compared using Kruskal-Wallis tests (B). Pearson correlation coefficients between SELL and various checkpoint molecules were calculated (C).

To further validate the results of our study, we used another independent dataset—an expression profile including the miRNA, lncRNA and genes of 12 HCC tumor tissues and paired paracancerous tissues (Shi et al., 2018)—for validating the expression alteration. Consistent with our results, LINC00261 (*P* = 6.60E−4, Fig. 6A) was decreased and MiR105-5p (*P* = 0.06, Fig. 6B) was increased in HCC samples. Meanwhile, SELL (*P* = 7.80E−2, Fig. 6C) was also decreased in HCC samples. However, values of SELL and MiR105-5p were not statistically significant (<0.05), which may be due to the small sample size. The Kaplan-Meier analyses and the Log-rank test were also used for validation in the GSE14520 datasets (Sun et al., 2017), including the expression profiles and clinical information of 221 HCC patients. In Fig. 6D, SELL had a significant positive association with OS (log-rank *P* = 2.50E−2). Due to the lack of miRNA and lncRNA expression profiles with clinical information, the association between OS and LINC00261 (or MiR105-5p) was not validated. The regulations and their binding sites between LINC00261, MiR105-5p and SELL were also predicted by Targetscan (Agarwal et al., 2015) and shown in Fig. 7A.

To further study the function of SELL, patients were divided into two groups according to SELL expression levels. The results, shown in Fig. 7B, suggested that patients with a higher expression level of SELL had a significantly increased fraction of naïve B cells, CD8+ T cells and M1 macrophages, and a decreased fraction of resting memory CD4+ T cells and activated NK cells. Meanwhile, a Pearson correlation coefficient was used for estimating the association between SELL and other checkpoint molecules (CD274, CTLA4, HAVCR2, LAG3, PDCD1) and Teff (effector T-cell) gene signatures (CD8A, CXCL6, CXCL10, EOMES, GZMA, GZMB, IFNG and TBX21) which have been reported as biomarkers related with cancer immunotherapy (Socinski et al., 2018). As shown in Fig. 7C, the expression level of SELL was significantly positively correlated with 12 known gene signatures (correlation coefficient r: 0.47–0.69) except for PDCD1 (r = 0.10). Taken together, these results indicated that SELL, regulated by MiR105-3p and LINC00261, affects the dysfunction of B cells and other immune cells. SELL is also associated with OS and may impact the efficacy of immunotherapy (not including PD1 or PDL1) in HCC.

## Discussion

During traditional chemotherapy for advanced HCC patients, the occurrence of drug-resistance is frequent which increases the rate of recurrence and metastasis (Ding et al., 2018). With the development of advanced experimental techniques including whole genome sequencing, RNA-Seq and CRISPR, many molecular mechanisms of the drug resistance regulation capability of ncRNAs have been uncovered. A signaling pathway, Hnf4α/MiR122/GALNT10, was found which could enhance the sensitivity of doxorubicin and sorafenib (Wu et al., 2015). Previous studies have also reported that MiR193b, MiR34a and MiR494 could increase the effect of sorafenib in HCC patients or cell lines by targeting various target genes (Mao et al., 2014; Wu et al., 2015; Yang et al., 2014). MiR216a and MiR217 could activate the PI3K/Akt and TGF-β signaling pathways for promoting drug resistance by targeting PTEN and SMAD7 (Xia, Ooi & Hui, 2013). Additionally, some studies found that lncRNA can participate in drug resistance through the regulation of cancer stem cells (Lv et al., 2018). The lncARSR could decrease the sensitivity of doxorubicin by increasing the expression level of PTEN and regulating the activation of the PI3K/Akt pathway (Li et al., 2017). Linc-VLDLR could improve sorafenib resistance by regulating the expression level of drug transporter genes (Takahashi et al., 2014). All of these findings suggest that miRNAs and lncRNAs are crucial regulators in the treatment of HCC.

Tang et al. (2019) have reported that the fraction of resting mast cells was higher in advanced HCC and significantly associated with patient outcomes. In the CheckMate-040 study, nivolumab plus ipilimumab was approved for patients with advanced HCC previously treated with sorafenib (Bristol Myers Squibb, 2020). The clinical results showed that 33 percent of patients responded to this combined treatment after 28 months of follow-up. However, the molecular mechanism used to identify HCC patients that may be sensitive to immunotherapy to avoid drug resistance is still unclear.

Our study focused on the dysfunction of TME in HCC patients. Our results found that the fractions of three subtypes of T cells, two subtypes of macrophages, memory B cells and five other types of immune cells were significantly altered in HCC tissues. A differentially expressed ceRNA network associated with OS was identified and a novel pathway, composed of LINC00261, MiR105-5p and SELL, was found. The alterations of these biomarkers were confirmed through independent datasets and SELL was also significantly positively associated with OS in an independent dataset of HCC patient samples. Interestingly, patients with a higher expression level of SELL also have a significantly increased fraction of naïve B cells, CD8+ T cells and M1 macrophages but also a decreased fraction of resting memory CD4+ T cells and activated NK cells. Recently, B cells associated with survival and immunotherapy response have been reported in sarcoma and melanoma (Cabrita et al., 2020; Petitprez et al., 2020).

The expression level of SELL was significantly positively correlated with 12 known gene signatures except for PDCD1 (r = 0.10). The most co-expressed genes were TBX21 (r = 0.69), EOMES (r = 0.66), CD8A (r = 0.64), GZMA (r = 0.64) and CXCL9 (r = 0.60). However, the roles of SELL in TME and HCC treatment were unclear. Additionally, out of the 10 immune related genes in our ceRNA network, seven genes (NR4A3 (nuclear receptor subfamily 4 group A member 3), SIK1 (salt inducible kinase 1), CD69, ADRB2 (adrenoceptor beta 2), GPR65 (G protein-coupled receptor 65), REPS2 (RALBP1 associated Eps domain containing 2)) were significantly decreased and positively associated with OS, and three genes (MYB (MYB proto-oncogene, transcription factor), CDC25A (cell division cycle 25A) and GPR19 (G protein-coupled receptor 19)) were significantly increased and negatively associated with OS. The known functions of these immune-related genes have been extensively studied. Wang et al. (2020) have reported that the restoration of NR4A3 can suppress the oncogenic roles of LINC00467 which enhances HCC cell proliferation. SIK1, which markedly suppresses epithelial-to-mesenchymal transition, tumor growth and lung metastasis through the WNT/β-catenin pathway (Qu et al., 2016), was significantly downregulated in HCC. ADRB2 has been reported as an important regulator to promote disease progression and sorafenib resistance by inhibiting autophagic degradation of HIF1α in HCC (Wu et al., 2016). GPR65 has been reported as a novel prognostic target for Glioblastoma (Yang et al., 2018a). The up-regulation of REPS2 is associated with nuclear DKK-1 expression and is correlated with decreased OS in colorectal cancer (Aguilera et al., 2015). Silencing CDC25A significantly inhibits the proliferation of liver cancer cells *in vitro* and *in vivo* (Chen et al., 2020). The overexpression of GPR19 can regulate E-cadherin expression and invasion of breast cancer cells (Rao & Herr, 2017). The roles of these genes in the occurrence and development of HCC need further study.

In our review of the literature, very few studies acknowledged SELL. Sun et al. (2008) reported that SELL significantly differentiates HCC patients with normal levels of alpha-fetoprotein (<20 ng/ml) from hepatitis patients. Meanwhile, a previous study found that MiR105 is overexpressed and positively associated with advanced TNM stage and poor overall survival in esophageal cancer cells (Gao et al., 2020). MiR105 is also downregulated in gastric cancer patients and cells. MiR105 effects the growth and aggressiveness of cancer cells by directly targeting SRY-box transcription factor 9 (Jin et al., 2019). Conversely, MiR105 is reduced in non-small cell lung cancer patients and significantly associated with poor overall survival and disease-free survival (Lu et al., 2017). The expression level of LINC00261 is decreased in non-small cell lung cancer and significantly correlated with lymphatic metastasis and survival time and suppressing the MiR105/FHL1 axis (Jin et al., 2019). In colon cancer, LINC00261, which has been identified as a tumor suppressor, can inhibit viability and proliferation capacity of cancer cells by sponging MiR324-3p and inactivating Wnt signaling (Yan et al., 2019). All of these studies on various cancers suggest both MiR105 and LINC00261 play important roles in cancer occurrence and progression.

Our study attempted to discover the differences of immune cells in HCC patients and to analyze the potential molecular mechanisms of those cells. However, there were some limitations to this early work. First, more algorithms and cutoff values could be used for prediction of the interactions of lncRNA-miRNA-gene and more immune-related signatures could be used for quantity analysis of the tumor immune environment. Second, it is difficult to find independent expression datasets that include the lncRNA, miRNA and genes of the same patients along with clinical information. The validation of all results should be carried out by laboratory testing in further studies. Despite these shortcomings, our study provided key insights into the roles of ncRNAs in the immunopathogenesis and possible clinical intervention of HCC.

## Conclusions

Although checkpoint inhibitors have improved the outcomes of HCC patients, drug resistance and a lower efficacy of immunotherapy still frequently occur increasing both recurrence and metastasis of HCC. The transcriptional regulation of SELL, significantly positively correlated with 12 known gene signatures of immunotherapy except for PDCD1, was affected by both LINC00261 and MiR105-5p. The results of this study may help identify potential ways to select novel biomarkers and improve immunotherapy efficacy in HCC.

## Supplemental Information

10.7717/peerj.12588/supp-1 Supplemental Information 1 The information of 337 HCC samples from TCGA.

10.7717/peerj.12588/supp-2 Supplemental Information 2 The GO BP and KEGG enrichments results of DEGs.

10.7717/peerj.12588/supp-3 Supplemental Information 3 The results of Kruskal-Wallis test between tumors and adjacent tissues.

10.7717/peerj.12588/supp-4 Supplemental Information 4 The 76 interactions in ceRNA network.

10.7717/peerj.12588/supp-5 Supplemental Information 5 The 58 biomarkers in the ceRNA network.

10.7717/peerj.12588/supp-6 Supplemental Information 6 The top 10% up-regulated and down-regulated DEGs.

## Additional Information and Declarations

### Data Availability

The following information was supplied regarding data availability: The gene, lncRNA, miRNA expression profiles, phenotypes, and survival data of the 379 TCGA samples (337 tumors and 42 adjacent tissues) are available at the UCSC Xena platform (https://xenabrowser.net/datapages/) at “GDC TCGA Liver Cancer (LIHC) (14 datasets)”. The independent datasets are available at GEO: GSE115019 and GSE14520.
