# Supplementary material for: The LINC00261/MiR105-5p/SELL axis is involved in dysfunction of B cell and is associated with overall survival in hepatocellular carcinoma
Source: PeerJ. 2022 Jun 9;10:e12588. doi: 10.7717/peerj.12588 (PMC9188773; doi:10.7717/peerj.12588)
Supplement: Supplemental Information 1 [file peerj-10-12588-s001.docx]

**Table S1. The information of 337 HCC samples from TCGA**

| Characteristics | Group | N(%) |
| --- | --- | --- |
| Age | <=60 | 165(49.00%) |
|  | >60 | 172(51.00%) |
| Sex | Male | 231(68.5%) |
|  | Female | 106(31.5%) |
| Race | White | 160(47.5%) |
|  | Asian | 153(45.4%) |
|  | Other | 24(7.1%) |
| TNM | I/II | 254(75.40%) |
|  | III/IV | 83(24.60%) |
| Child-Pugh grade | A | 205(60.80%) |
|  | B | 21(6.20%) |
|  | C | 1(0.30%) |
|  | Unknown | 111(32.90%) |
| Fibrosis ishak score | 0 - No Fibrosis | 69(20.50%) |
|  | 1,2 - Portal Fibrosis | 28(8.30%) |
|  | 3,4 - Fibrous Speta | 25(7.40%) |
|  | 5 - Nodular Formation and Incomplete Cirrhosis | 8(2.40%) |
|  | 6 - Established Cirrhosis | 66(19.60%) |
|  | Unknown | 141(41.80%) |
