# Supplementary material for: The LINC00261/MiR105-5p/SELL axis is involved in dysfunction of B cell and is associated with overall survival in hepatocellular carcinoma
Source: PeerJ. 2022 Jun 9;10:e12588. doi: 10.7717/peerj.12588 (PMC9188773; doi:10.7717/peerj.12588)
Supplement: Supplemental Information 2 [file peerj-10-12588-s002.docx]

Table S2. The GO BP and KEGG enrichments results of DEGs

| DEGs | Category | Term | Q value |
| --- | --- | --- | --- |
| Up | GO BP | cell division | 1.38E-11 |
| Up | KEGG | Cell cycle | 8.69E-11 |
| Up | GO BP | G1/S transition of mitotic cell cycle | 5.23E-09 |
| Up | GO BP | DNA replication | 5.81E-09 |
| Up | GO BP | sister chromatid cohesion | 6.99E-09 |
| Up | GO BP | DNA replication initiation | 2.52E-07 |
| Up | GO BP | mitotic nuclear division | 4.36E-07 |
| Up | KEGG | DNA replication | 6.56E-07 |
| Up | GO BP | mitotic sister chromatid segregation | 1.14E-04 |
| Up | GO BP | microtubule-based movement | 4.57E-04 |
| Up | GO BP | mitotic cytokinesis | 8.70E-04 |
| Up | GO BP | DNA strand elongation involved in DNA replication | 8.79E-03 |
| Up | GO BP | chromosome segregation | 1.22E-02 |
| Up | GO BP | CENP-A containing nucleosome assembly | 1.78E-02 |
| Up | GO BP | telomere maintenance via recombination | 2.36E-02 |
| Up | GO BP | embryonic skeletal system morphogenesis | 3.33E-02 |
| Up | GO BP | G2/M transition of mitotic cell cycle | 3.48E-02 |
| Up | GO BP | cell proliferation | 3.89E-02 |
| Up | KEGG | Small cell lung cancer | 8.32E-03 |
| Up | KEGG | ECM-receptor interaction | 1.14E-02 |
| Down | KEGG | Complement and coagulation cascades | 1.66E-21 |
| Down | KEGG | Chemical carcinogenesis | 8.31E-17 |
| Down | KEGG | Metabolic pathways | 2.08E-16 |
| Down | KEGG | Drug metabolism - cytochrome P450 | 1.27E-15 |
| Down | GO BP | xenobiotic metabolic process | 4.27E-14 |
| Down | GO BP | oxidation-reduction process | 1.94E-13 |
| Down | GO BP | platelet degranulation | 5.57E-13 |
| Down | GO BP | acute-phase response | 3.34E-12 |
| Down | KEGG | Metabolism of xenobiotics by cytochrome P450 | 3.96E-12 |
| Down | GO BP | inflammatory response | 9.81E-11 |
| Down | GO BP | metabolic process | 1.72E-10 |
| Down | KEGG | Retinol metabolism | 2.49E-10 |
| Down | GO BP | epoxygenase P450 pathway | 4.60E-09 |
| Down | GO BP | response to drug | 5.78E-09 |
| Down | GO BP | cell adhesion | 2.42E-07 |
| Down | GO BP | drug metabolic process | 2.44E-07 |
| Down | KEGG | Steroid hormone biosynthesis | 1.98E-08 |
| Down | KEGG | Tryptophan metabolism | 8.72E-08 |
| Down | KEGG | Staphylococcus aureus infection | 1.03E-07 |
| Down | KEGG | Fatty acid degradation | 3.05E-07 |
| Down | GO BP | steroid metabolic process | 7.84E-06 |
| Down | GO BP | cytokine-mediated signaling pathway | 1.05E-05 |
| Down | KEGG | Valine, leucine and isoleucine degradation | 7.24E-07 |
| Down | GO BP | negative regulation of endopeptidase activity | 1.30E-05 |
| Down | GO BP | response to lipopolysaccharide | 2.89E-05 |
| Down | KEGG | Drug metabolism - other enzymes | 2.78E-06 |
| Down | KEGG | Glycine, serine and threonine metabolism | 2.86E-06 |
| Down | GO BP | cell chemotaxis | 5.47E-05 |
| Down | GO BP | complement activation, alternative pathway | 1.43E-04 |
| Down | GO BP | receptor-mediated endocytosis | 1.85E-04 |
| Down | GO BP | immune response | 2.28E-04 |
| Down | KEGG | Tyrosine metabolism | 1.49E-05 |
| Down | GO BP | cellular amino acid metabolic process | 6.91E-04 |
| Down | GO BP | bile acid biosynthetic process | 8.52E-04 |
| Down | GO BP | fibrinolysis | 8.52E-04 |
| Down | KEGG | Ascorbate and aldarate metabolism | 5.20E-05 |
| Down | KEGG | Butanoate metabolism | 5.20E-05 |
| Down | KEGG | Bile secretion | 5.45E-05 |
| Down | GO BP | blood coagulation | 1.05E-03 |
| Down | GO BP | flavonoid glucuronidation | 1.70E-03 |
| Down | KEGG | PPAR signaling pathway | 1.17E-04 |
| Down | GO BP | cell surface receptor signaling pathway | 2.13E-03 |
| Down | GO BP | negative regulation of fibrinolysis | 2.36E-03 |
| Down | KEGG | Biosynthesis of antibiotics | 1.58E-04 |
| Down | GO BP | negative regulation of JAK-STAT cascade | 4.35E-03 |
| Down | GO BP | cholesterol homeostasis | 4.44E-03 |
| Down | GO BP | flavonoid biosynthetic process | 4.76E-03 |
| Down | GO BP | positive regulation of ERK1 and ERK2 cascade | 1.33E-02 |
| Down | GO BP | reverse cholesterol transport | 1.34E-02 |
| Down | GO BP | blood coagulation, intrinsic pathway | 1.34E-02 |
| Down | GO BP | lipid metabolic process | 1.59E-02 |
| Down | GO BP | triglyceride homeostasis | 1.69E-02 |
| Down | GO BP | cellular amino acid biosynthetic process | 1.69E-02 |
| Down | KEGG | Mineral absorption | 1.06E-03 |
| Down | GO BP | fatty acid beta-oxidation | 1.92E-02 |
| Down | KEGG | Carbon metabolism | 1.16E-03 |
| Down | GO BP | chemotaxis | 2.32E-02 |
| Down | GO BP | cellular response to tumor necrosis factor | 2.34E-02 |
| Down | GO BP | response to glucocorticoid | 2.45E-02 |
| Down | GO BP | cellular response to zinc ion | 2.55E-02 |
| Down | KEGG | Pentose and glucuronate interconversions | 1.44E-03 |
| Down | GO BP | chemokine-mediated signaling pathway | 2.87E-02 |
| Down | GO BP | glutathione metabolic process | 3.63E-02 |
| Down | KEGG | Prion diseases | 2.27E-03 |
| Down | GO BP | cellular response to lipopolysaccharide | 4.04E-02 |
| Down | GO BP | positive regulation of synapse assembly | 4.44E-02 |
| Down | GO BP | positive regulation of inflammatory response | 4.61E-02 |
| Down | KEGG | Arginine biosynthesis | 2.72E-03 |
| Down | KEGG | Primary bile acid biosynthesis | 3.15E-03 |
| Down | KEGG | beta-Alanine metabolism | 3.27E-03 |
| Down | KEGG | Alanine, aspartate and glutamate metabolism | 3.49E-03 |
| Down | KEGG | Malaria | 6.26E-03 |
| Down | KEGG | Histidine metabolism | 8.86E-03 |
| Down | KEGG | Porphyrin and chlorophyll metabolism | 1.06E-02 |
| Down | KEGG | Cytokine-cytokine receptor interaction | 2.36E-02 |
| Down | KEGG | Propanoate metabolism | 2.59E-02 |
| Down | KEGG | Arachidonic acid metabolism | 4.87E-02 |
