# Supplementary material for: The LINC00261/MiR105-5p/SELL axis is involved in dysfunction of B cell and is associated with overall survival in hepatocellular carcinoma
Source: PeerJ. 2022 Jun 9;10:e12588. doi: 10.7717/peerj.12588 (PMC9188773; doi:10.7717/peerj.12588)
Supplement: Supplemental Information 3 [file peerj-10-12588-s003.docx]

**Table S3. The results of Kruskal-Wallis test between tumors and adjacent tissues**

| Cell type | P value | Adjusted p value |
| --- | --- | --- |
| B cells naive | 6.49E-01 | 6.50E-01 |
| B cells memory | 2.07E-02 | 2.10E-02 |
| Plasma cells | 1.98E-02 | 2.00E-02 |
| T cells CD8 | 4.79E-01 | 4.80E-01 |
| T cells CD4 naive | 9.28E-01 | 9.30E-01 |
| T cells CD4 memory resting | 4.90E-03 | 4.90E-03 |
| T cells CD4 memory activated | 2.22E-01 | 2.20E-01 |
| T cells follicular helper | 2.28E-02 | 2.30E-02 |
| T cells regulatory(Tregs) | 6.74E-13 | 6.70E-13 |
| T cells gamma delta | 3.74E-01 | 3.70E-01 |
| NK cells resting | 1.58E-02 | 1.60E-02 |
| NK cells activated | 7.27E-01 | 7.30E-01 |
| Monocytes | 5.23E-08 | 5.20E-08 |
| Macrophages M0 | 2.17E-09 | 2.20E-09 |
| Macrophages M1 | 5.35E-01 | 5.40E-01 |
| Macrophages M2 | 2.16E-03 | 2.20E-03 |
| Dendritic cells resting | 8.75E-03 | 8.70E-03 |
| Dendritic cells activated | 8.66E-01 | 8.70E-01 |
| Mast cells resting | 8.15E-01 | 8.20E-01 |
| Mast cells activated | NA | NA |
| Eosinophils | NA | NA |
| Neutrophils | 2.97E-04 | 3.00E-04 |
