# Supplementary material for: The LINC00261/MiR105-5p/SELL axis is involved in dysfunction of B cell and is associated with overall survival in hepatocellular carcinoma
Source: PeerJ. 2022 Jun 9;10:e12588. doi: 10.7717/peerj.12588 (PMC9188773; doi:10.7717/peerj.12588)
Supplement: Supplemental Information 4 [file peerj-10-12588-s004.docx]

**Table S4. The 76 interactions in ceRNA network**

| Biomarker 1 | Biomarker 2 | Interaction type |
| --- | --- | --- |
| let7c-5p | AC020915.3 | miR2LncRNA |
| let7c-5p | ADRB2 | miR2Gene |
| let7c-5p | CDC25A | miR2Gene |
| let7c-5p | LINC00665 | miR2LncRNA |
| let7c-5p | SNHG12 | miR2LncRNA |
| miR105-5p | AL049840.4 | miR2LncRNA |
| miR105-5p | CYTOR | miR2LncRNA |
| miR105-5p | LINC00261 | miR2LncRNA |
| miR105-5p | MIR4435-2HG | miR2LncRNA |
| miR105-5p | MYB | miR2Gene |
| miR105-5p | RUSC1-AS1 | miR2LncRNA |
| miR105-5p | SELL | miR2Gene |
| miR105-5p | SNHG7 | miR2LncRNA |
| miR10b-5p | NR4A3 | miR2Gene |
| miR10b-5p | SNHG3 | miR2LncRNA |
| miR10b-5p | SNHG7 | miR2LncRNA |
| miR1301-3p | DANCR | miR2LncRNA |
| miR1301-3p | NR4A3 | miR2Gene |
| miR1301-3p | PVT1 | miR2LncRNA |
| miR1301-3p | PXN-AS1 | miR2LncRNA |
| miR1301-3p | SNHG12 | miR2LncRNA |
| miR1301-3p | SNHG7 | miR2LncRNA |
| miR1301-3p | WAC-AS1 | miR2LncRNA |
| miR148a-3p | AC073896.4 | miR2LncRNA |
| miR148a-3p | AL031673.1 | miR2LncRNA |
| miR148a-3p | AL049840.4 | miR2LncRNA |
| miR148a-3p | LINC01554 | miR2LncRNA |
| miR148a-3p | PVT1 | miR2LncRNA |
| miR148a-3p | SIK1 | miR2Gene |
| miR148a-3p | SNHG20 | miR2LncRNA |
| miR148a-3p | SNHG3 | miR2LncRNA |
| miR25-3p | CD69 | miR2Gene |
| miR25-3p | NR4A3 | miR2Gene |
| miR25-3p | PITPNA-AS1 | miR2LncRNA |
| miR25-3p | SNHG17 | miR2LncRNA |
| miR29c-3p | AC012146.1 | miR2LncRNA |
| miR29c-3p | AL355488.1 | miR2LncRNA |
| miR29c-3p | ARRDC1-AS1 | miR2LncRNA |
| miR29c-3p | CRNDE | miR2LncRNA |
| miR29c-3p | EFNA5 | miR2Gene |
| miR29c-3p | PVT1 | miR2LncRNA |
| miR29c-3p | REPS2 | miR2Gene |
| miR29c-3p | SNHG17 | miR2LncRNA |
| miR29c-3p | SNHG20 | miR2LncRNA |
| miR29c-3p | THUMPD3-AS1 | miR2LncRNA |
| miR301a-3p | AL049840.4 | miR2LncRNA |
| miR301a-3p | AP003469.4 | miR2LncRNA |
| miR301a-3p | CD69 | miR2Gene |
| miR301b-3p | AL049840.4 | miR2LncRNA |
| miR301b-3p | AP003469.4 | miR2LncRNA |
| miR301b-3p | CD69 | miR2Gene |
| miR3200-3p | AC132872.1 | miR2LncRNA |
| miR3200-3p | DANCR | miR2LncRNA |
| miR3200-3p | REPS2 | miR2Gene |
| miR3200-3p | U62317.3 | miR2LncRNA |
| miR421 | MINCR | miR2LncRNA |
| miR421 | REPS2 | miR2Gene |
| miR421 | SNHG1 | miR2LncRNA |
| miR421 | SNHG17 | miR2LncRNA |
| miR421 | THUMPD3-AS1 | miR2LncRNA |
| miR421 | ZFAS1 | miR2LncRNA |
| miR454-3p | AL049840.4 | miR2LncRNA |
| miR454-3p | AP003469.4 | miR2LncRNA |
| miR454-3p | CD69 | miR2Gene |
| miR454-3p | GPR65 | miR2Gene |
| miR9-3p | AL355488.1 | miR2LncRNA |
| miR9-3p | GPR19 | miR2Gene |
| miR9-3p | LINC00261 | miR2LncRNA |
| miR9-3p | SNHG1 | miR2LncRNA |
| miR9-5p | CRNDE | miR2LncRNA |
| miR9-5p | LINC00665 | miR2LncRNA |
| miR9-5p | RAB30-AS1 | miR2LncRNA |
| miR9-5p | SIK1 | miR2Gene |
| miR9-5p | SNHG1 | miR2LncRNA |
| miR9-5p | SNHG7 | miR2LncRNA |
| miR9-5p | THUMPD3-AS1 | miR2LncRNA |
