# Supplementary material for: The LINC00261/MiR105-5p/SELL axis is involved in dysfunction of B cell and is associated with overall survival in hepatocellular carcinoma
Source: PeerJ. 2022 Jun 9;10:e12588. doi: 10.7717/peerj.12588 (PMC9188773; doi:10.7717/peerj.12588)
Supplement: Supplemental Information 5 [file peerj-10-12588-s005.docx]

**Table S5. The 58 biomarkers in the ceRNA network**

| name1 | HR(95%CI) | LOGFC | Type |
| --- | --- | --- | --- |
| ADRB2 | 0.889(0.803-0.984) | -1.42 | Gene |
| CD69 | 0.883(0.797-0.979) | -1.60 | Gene |
| CDC25A | 1.3(1.14-1.48) | 1.83 | Gene |
| EFNA5 | 1.09(1.02-1.17) | -1.13 | Gene |
| GPR19 | 1.14(1-1.3) | 1.97 | Gene |
| GPR65 | 0.838(0.72-0.977) | -1.31 | Gene |
| MYB | 1.14(1.02-1.28) | 1.08 | Gene |
| NR4A3 | 0.893(0.799-1) | -2.56 | Gene |
| REPS2 | 0.783(0.694-0.883) | -1.09 | Gene |
| SELL | 0.836(0.732-0.954) | -1.14 | Gene |
| SIK1 | 0.869(0.761-0.992) | -1.63 | Gene |
| AC012146.1 | 1.4(1.12-1.76) | 0.74 | LncRNA |
| AC020915.3 | 1.98(1.35-2.91) | 0.59 | LncRNA |
| AC073896.4 | 1.33(1.1-1.62) | 0.61 | LncRNA |
| AC132872.1 | 1.33(1.01-1.76) | 0.81 | LncRNA |
| AL031673.1 | 1.37(1.04-1.82) | 0.78 | LncRNA |
| AL049840.4 | 1.48(1.14-1.93) | 0.99 | LncRNA |
| AL355488.1 | 1.48(1.1-1.99) | 0.66 | LncRNA |
| AP003469.4 | 1.39(1.08-1.8) | 0.78 | LncRNA |
| ARRDC1-AS1 | 1.57(1.11-2.21) | 0.75 | LncRNA |
| CRNDE | 1.24(1-1.53) | 0.96 | LncRNA |
| CYTOR | 1.38(1.16-1.63) | 1.40 | LncRNA |
| DANCR | 1.34(1.14-1.58) | 0.89 | LncRNA |
| LINC00261 | 0.848(0.726-0.991) | -0.90 | LncRNA |
| LINC00665 | 1.34(1.09-1.64) | 0.77 | LncRNA |
| LINC01554 | 0.883(0.809-0.964) | -1.18 | LncRNA |
| MINCR | 1.29(1.07-1.54) | 1.04 | LncRNA |
| MIR4435-2HG | 1.51(1.18-1.94) | 1.03 | LncRNA |
| PITPNA-AS1 | 1.32(1.03-1.7) | 0.84 | LncRNA |
| PVT1 | 1.39(1.06-1.82) | 0.66 | LncRNA |
| PXN-AS1 | 1.79(1.27-2.53) | 0.66 | LncRNA |
| RAB30-AS1 | 1.63(1.13-2.36) | 0.65 | LncRNA |
| RUSC1-AS1 | 1.38(1.03-1.86) | 0.93 | LncRNA |
| SNHG1 | 1.43(1.14-1.8) | 1.51 | LncRNA |
| SNHG12 | 1.57(1.19-2.08) | 0.85 | LncRNA |
| SNHG17 | 1.3(1.03-1.65) | 0.95 | LncRNA |
| SNHG20 | 1.82(1.24-2.68) | 0.69 | LncRNA |
| SNHG3 | 1.54(1.28-1.85) | 1.11 | LncRNA |
| SNHG7 | 1.28(1.04-1.57) | 1.36 | LncRNA |
| THUMPD3-AS1 | 2.04(1.42-2.95) | 0.71 | LncRNA |
| U62317.3 | 1.36(1.06-1.75) | 0.80 | LncRNA |
| WAC-AS1 | 1.77(1.3-2.41) | 0.68 | LncRNA |
| ZFAS1 | 1.22(1.02-1.45) | 1.22 | LncRNA |
| let-7c-5p | 0.862(0.748-0.993) | -1.69 | miRNA |
| miR105-5p | 1.14(1.07-1.21) | 1.60 | miRNA |
| miR10b-5p | 1.15(1.04-1.28) | 2.75 | miRNA |
| miR1301-3p | 1.32(1.1-1.58) | 0.88 | miRNA |
| miR148a-3p | 0.807(0.681-0.958) | -0.72 | miRNA |
| miR25-3p | 1.46(1.14-1.88) | 0.61 | miRNA |
| miR29c-3p | 0.804(0.665-0.971) | -1.44 | miRNA |
| miR301a-3p | 1.4(1.18-1.67) | 0.71 | miRNA |
| miR301b-3p | 1.32(1.1-1.59) | 0.61 | miRNA |
| miR3200-3p | 1.23(1.08-1.41) | 0.89 | miRNA |
| miR421 | 1.57(1.26-1.96) | 0.67 | miRNA |
| miR454-3p | 1.42(1.14-1.78) | 0.66 | miRNA |
| miR9-3p | 1.17(1.09-1.27) | 1.22 | miRNA |
| miR9-5p | 1.17(1.09-1.27) | 1.22 | miRNA |
