# Supplementary material for: The LINC00261/MiR105-5p/SELL axis is involved in dysfunction of B cell and is associated with overall survival in hepatocellular carcinoma
Source: PeerJ. 2022 Jun 9;10:e12588. doi: 10.7717/peerj.12588 (PMC9188773; doi:10.7717/peerj.12588)
Supplement: Supplemental Information 6 [file peerj-10-12588-s006.docx]

**Table S6.The top 10% up-regulated and down-regulated DEGs**

| Gene | LOG2FC | AdjPValue | Gene | LOG2FC | AdjPValue |
| --- | --- | --- | --- | --- | --- |
| CLEC4M | -8.27 | 5.48E-113 | **HEPACAM** | -3.84 | 6.54E-78 |
| CLEC4G | -8.12 | 3.26E-131 | **RANBP3L** | -3.82 | 7.92E-110 |
| INS-IGF2 | -7.89 | 4.12E-97 | **TMEM27** | -3.80 | 2.54E-193 |
| CLEC1B | -7.80 | 8.56E-101 | **PROM1** | -3.79 | 2.47E-77 |
| CYP1A2 | -7.73 | 1.6E-123 | **PLAC8** | -3.79 | 2.23E-151 |
| GDF2 | -7.59 | 1.16E-97 | **CYP2B6** | -3.79 | 6.4E-248 |
| FCN2 | -7.42 | 8.47E-124 | **PCK1** | -3.78 | 4.47E-249 |
| MARCO | -7.38 | 2.97E-124 | **DNASE1L3** | -3.78 | 9.07E-248 |
| STAB2 | -7.17 | 2.45E-117 | **GYS2** | -3.77 | 6.68E-197 |
| MT1H | -7.01 | 8.3E-112 | **LOC255167** | -3.77 | 4.75E-155 |
| HAMP | -6.87 | 1.74E-146 | **FAM134B** | -3.76 | 3.38E-187 |
| PZP | -6.81 | 2.95E-103 | **FOS** | -3.76 | 4.07E-294 |
| CRHBP | -6.52 | 2.18E-158 | **GABRB3** | -3.75 | 2.01E-75 |
| CXCL14 | -6.51 | 3.66E-119 | **CCL23** | -3.75 | 2.09E-127 |
| MT1G | -6.42 | 6.04E-171 | **SRD5A2** | -3.75 | 6.94E-178 |
| SFRP5 | -6.12 | 2.79E-81 | **ANGPTL6** | -3.74 | 1.75E-227 |
| CNDP1 | -5.96 | 6.37E-109 | **PITPNM3** | -3.74 | 3.55E-160 |
| COLEC10 | -5.87 | 1.49E-147 | **KCNJ16** | -3.73 | 6.27E-71 |
| FCN3 | -5.83 | 3E-200 | **LYVE1** | -3.71 | 1.73E-236 |
| BMPER | -5.77 | 4.32E-137 | **GNAO1** | -3.70 | 3.1E-177 |
| CYP3A4 | -5.75 | 4.62E-176 | **GBA3** | -3.69 | 6.67E-196 |
| MT1M | -5.69 | 1.27E-143 | **HAND2** | -3.69 | 3.52E-121 |
| C9 | -5.60 | 3.33E-167 | **NTF3** | -3.68 | 2.79E-149 |
| CHST4 | -5.60 | 9.59E-83 | **AADAT** | -3.68 | 3.76E-245 |
| HSD17B13 | -5.52 | 2.05E-159 | **SLCO4C1** | -3.68 | 1.11E-111 |
| MT1F | -5.44 | 5.95E-185 | **ECM1** | -3.68 | 5.23E-281 |
| TTC36 | -5.43 | 1.51E-137 | **MT2A** | -3.66 | 9.87E-305 |
| SLCO1B3 | -5.42 | 1.87E-121 | **ST6GAL2** | -3.66 | 2E-119 |
| SYT9 | -5.18 | 2.86E-72 | **CYP8B1** | -3.65 | 5.26E-209 |
| C14orf68 | -5.16 | 1.24E-185 | **IGF2** | -3.63 | 5.98E-195 |
| CYP26A1 | -5.16 | 2.33E-120 | **CXCL12** | -3.62 | 6.27E-283 |
| HHIP | -5.14 | 1.38E-93 | **FCGR2B** | -3.62 | 1.16E-178 |
| C14orf180 | -5.09 | 2.48E-88 | **IGJ** | -3.60 | 1.55E-166 |
| FREM2 | -5.08 | 1.59E-83 | **RAB25** | -3.60 | 2.09E-59 |
| MUC6 | -5.06 | 9.31E-90 | **SLC3A1** | -3.58 | 1.49E-130 |
| CD5L | -5.04 | 8.92E-150 | **ANXA8** | -3.57 | 8.41E-87 |
| SLC5A1 | -5.03 | 6.21E-84 | **PRSS8** | -3.57 | 4.76E-121 |
| IGFALS | -4.97 | 5.42E-194 | **CAMK2B** | -3.57 | 1.88E-124 |
| KCNN2 | -4.90 | 3.66E-146 | **SRPX** | -3.57 | 3.12E-170 |
| CCBE1 | -4.86 | 4.64E-110 | **NNMT** | -3.57 | 9.76E-253 |
| CFTR | -4.84 | 4.08E-75 | **KBTBD11** | -3.56 | 6.75E-192 |
| SLC22A1 | -4.83 | 8.55E-195 | **AKR1D1** | -3.56 | 4.06E-207 |
| NAT2 | -4.81 | 1.12E-154 | **BBOX1** | -3.56 | 2.04E-162 |
| MT1E | -4.77 | 1.37E-215 | **FAM65C** | -3.53 | 2.38E-187 |
| UROC1 | -4.76 | 3.52E-160 | **FAM99B** | -3.53 | 2.16E-94 |
| RSPO3 | -4.75 | 3.06E-110 | **CALN1** | -3.51 | 1.5E-77 |
| THRSP | -4.73 | 3.24E-138 | **INMT** | -3.51 | 4.16E-224 |
| HAO2 | -4.72 | 3.9E-170 | **CYP1A1** | -3.50 | 4.85E-128 |
| MFSD2A | -4.72 | 1.08E-172 | **LIFR** | -3.50 | 2.07E-269 |
| ADRA1A | -4.71 | 8.9E-148 | **MME** | -3.49 | 2.42E-120 |
| VIPR1 | -4.71 | 6.16E-194 | **RET** | -3.49 | 1.47E-112 |
| ASPG | -4.64 | 1.86E-138 | **SLC10A1** | -3.48 | 2.31E-198 |
| DBH | -4.63 | 3.52E-201 | **LCAT** | -3.48 | 4.26E-287 |
| CDHR2 | -4.62 | 1.42E-131 | **TSLP** | -3.47 | 2.06E-152 |
| CYP2A7 | -4.62 | 3.05E-115 | **KCND3** | -3.47 | 1.89E-120 |
| HGFAC | -4.61 | 1.61E-149 | **MT1L** | -3.46 | 1.98E-168 |
| CLDN10 | -4.60 | 3.31E-72 | **BMP5** | -3.45 | 2.13E-81 |
| BCO2 | -4.60 | 1.34E-183 | **SLITRK6** | -3.45 | 6.75E-77 |
| GPM6A | -4.54 | 1E-146 | **MFAP4** | -3.43 | 3.55E-165 |
| SLITRK3 | -4.52 | 1.39E-69 | **RDH16** | -3.42 | 2.64E-246 |
| TIMD4 | -4.51 | 3.5E-104 | **SPP2** | -3.40 | 1.38E-196 |
| CYP2A6 | -4.49 | 8.68E-185 | **OXT** | -3.39 | 1.48E-65 |
| FOSB | -4.44 | 9.93E-222 | **NPY1R** | -3.38 | 9.77E-195 |
| GLS2 | -4.43 | 8.82E-173 | **GREM2** | -3.38 | 7.33E-145 |
| APOF | -4.37 | 5.71E-224 | **PDGFRA** | -3.38 | 1.03E-171 |
| PTH1R | -4.35 | 1.33E-205 | **NRG1** | -3.38 | 7.55E-108 |
| BMP10 | -4.33 | 2.05E-93 | **HBA1** | -3.37 | 2.69E-147 |
| C7 | -4.30 | 3.08E-190 | **ANKRD55** | -3.37 | 1.05E-134 |
| NDST3 | -4.30 | 1.21E-103 | **SLC34A2** | -3.36 | 2.34E-73 |
| ADH4 | -4.29 | 2.44E-220 | **CNTN3** | -3.34 | 9.18E-89 |
| SLC6A19 | -4.23 | 2.66E-64 | **SLC22A10** | -3.34 | 1.98E-204 |
| GPR182 | -4.23 | 1.95E-119 | **CDH19** | -3.33 | 5.67E-90 |
| CYP39A1 | -4.23 | 1.47E-179 | **IDO2** | -3.33 | 5.11E-102 |
| FAM180A | -4.22 | 1.85E-122 | **HGF** | -3.33 | 2.78E-165 |
| DPT | -4.21 | 1.68E-130 | **B3GAT1** | -3.32 | 1.2E-115 |
| CYP2E1 | -4.21 | 1.26E-214 | **F9** | -3.32 | 2.13E-231 |
| DCN | -4.19 | 2.86E-209 | **AMHR2** | -3.31 | 1.04E-81 |
| GCGR | -4.18 | 1.61E-146 | **UGT1A4** | -3.30 | 2.43E-191 |
| MOGAT2 | -4.16 | 4.01E-140 | **TDO2** | -3.30 | 3.16E-247 |
| FOLH1B | -4.15 | 2.5E-114 | **TAT** | -3.29 | 3.34E-228 |
| NBLA00301 | -4.14 | 1.12E-106 | **FAM198A** | -3.29 | 5.99E-153 |
| ITLN1 | -4.13 | 4.09E-91 | **CYP4A11** | -3.29 | 2.64E-278 |
| CYP2C8 | -4.12 | 2.48E-248 | **CYP2C19** | -3.29 | 4.77E-215 |
| MT1X | -4.10 | 2.22E-247 | **HPGD** | -3.28 | 1.08E-176 |
| IL13RA2 | -4.10 | 2.82E-103 | **CETP** | -3.28 | 1.28E-249 |
| CFP | -4.07 | 1.42E-241 | **ADH1C** | -3.28 | 5.4E-237 |
| FAM99A | -4.04 | 1.59E-134 | **TRPV6** | -3.27 | 7.85E-67 |
| ESR1 | -4.03 | 1.02E-188 | **CNTFR** | -3.27 | 9.52E-110 |
| GLYAT | -4.02 | 1.12E-178 | **SAA2** | -3.27 | 3.18E-176 |
| LRAT | -4.00 | 1.98E-189 | **CLIC6** | -3.25 | 3.2E-102 |
| OIT3 | -3.99 | 6.4E-269 | **KRT19** | -3.25 | 2.59E-99 |
| AVPR1A | -3.98 | 5.38E-108 | **VTCN1** | -3.25 | 2.44E-77 |
| DIRAS3 | -3.96 | 1.51E-151 | **TMEM82** | -3.24 | 5E-196 |
| LPA | -3.95 | 6.81E-219 | **PLCXD3** | -3.23 | 2.53E-126 |
| SFRP1 | -3.93 | 2.11E-142 | **SHBG** | -3.22 | 3.76E-212 |
| COL6A6 | -3.91 | 1.16E-106 | **KANK4** | -3.22 | 2.2E-107 |
| GABRP | -3.91 | 2.47E-89 | **HSD11B1** | -3.22 | 1.84E-216 |
| FAM151A | -3.91 | 4.45E-107 | **TPPP2** | -3.19 | 4.33E-129 |
| GPR128 | -3.90 | 3.34E-189 | **FXYD1** | -3.19 | 8.53E-210 |
| FAM83F | -3.90 | 9.35E-77 | **MUM1L1** | -3.18 | 4.14E-88 |
| PVALB | -3.90 | 1.39E-99 | **C19orf77** | -3.18 | 2.29E-105 |
| CLRN3 | -3.89 | 7.1E-135 | **LY6E** | -3.18 | 1.99E-284 |
| HEPN1 | -3.85 | 2.35E-71 | **CYP2C9** | -3.18 | 2.31E-265 |
| SCARA5 | -3.85 | 4.13E-86 | **ADAMTS13** | -3.18 | 0 |
| THBS4 | 5.46 | 6.44E-172 | **PRC1** | 3.32 | 0 |
| ZIC2 | 5.01 | 5.34E-141 | **KIF15** | 3.31 | 2.82E-186 |
| GPC3 | 4.90 | 8.18E-226 | **CCNA2** | 3.30 | 2.37E-276 |
| IGF2BP1 | 4.75 | 1.06E-101 | **CDT1** | 3.30 | 2.67E-272 |
| EPS8L3 | 4.74 | 1.55E-142 | **DTL** | 3.29 | 3.37E-270 |
| CPLX2 | 4.67 | 9.86E-78 | **MKI67** | 3.27 | 3.69E-299 |
| NUF2 | 4.50 | 2.55E-227 | **AKR1B10** | 3.27 | 3.2E-180 |
| CDC25C | 4.41 | 5.95E-215 | **CENPE** | 3.27 | 2.89E-243 |
| ESM1 | 4.40 | 6.08E-240 | **CDCA5** | 3.27 | 5.35E-295 |
| GABRD | 4.38 | 6.52E-248 | **EEF1A2** | 3.25 | 7.02E-125 |
| BIRC5 | 4.28 | 1.13E-237 | **SSX1** | 3.25 | 2.24E-45 |
| CDC20 | 4.27 | 1.59E-253 | **DIAPH3** | 3.24 | 6.38E-210 |
| CDKN3 | 4.25 | 4.94E-252 | **HOXD9** | 3.23 | 3.07E-127 |
| UBE2C | 4.21 | 4.01E-252 | **RRM2** | 3.23 | 8.89E-308 |
| TROAP | 4.19 | 5.39E-234 | **GTSE1** | 3.22 | 4.04E-229 |
| TOP2A | 4.16 | 1.53E-286 | **MMP11** | 3.22 | 2.13E-239 |
| KIF20A | 4.16 | 1.75E-245 | **UHRF1** | 3.22 | 6.16E-236 |
| COL15A1 | 4.15 | 4.14E-282 | **C15orf42** | 3.21 | 1.17E-205 |
| IQGAP3 | 4.14 | 3.21E-264 | **RAD54L** | 3.21 | 1.55E-207 |
| CCNB2 | 4.13 | 1.91E-252 | **CELSR3** | 3.21 | 2.11E-241 |
| SKA1 | 4.10 | 4.3E-228 | **CTHRC1** | 3.19 | 2.92E-170 |
| MELK | 4.10 | 2.71E-245 | **COX4I2** | 3.19 | 2.49E-205 |
| MYBL2 | 4.09 | 1.03E-232 | **SULT1C2** | 3.17 | 5.2E-144 |
| NEK2 | 4.08 | 1.27E-242 | **HOXA10** | 3.15 | 4.91E-96 |
| TGM3 | 4.08 | 3.77E-142 | **NCAPH** | 3.14 | 8.63E-250 |
| KIF4A | 4.07 | 1.66E-249 | **FBXO43** | 3.14 | 5.02E-200 |
| TTK | 4.06 | 1.81E-210 | **SPC24** | 3.14 | 2.17E-178 |
| MAGEA1 | 4.05 | 5.73E-66 | **DUSP9** | 3.14 | 4.95E-190 |
| HOXA13 | 4.05 | 1.05E-101 | **CCNB1** | 3.13 | 0 |
| DLGAP5 | 4.01 | 3.97E-227 | **MAGEC2** | 3.12 | 1.84E-49 |
| NCAPG | 4.00 | 7.98E-273 | **MCM10** | 3.12 | 6.75E-212 |
| KIF2C | 3.98 | 4.42E-242 | **AIM1L** | 3.11 | 7.11E-186 |
| ISX | 3.91 | 1.42E-80 | **COL7A1** | 3.09 | 4.53E-159 |
| BUB1 | 3.87 | 5.06E-237 | **LYPD1** | 3.08 | 2.07E-173 |
| CDC45 | 3.87 | 2.05E-233 | **CDCA8** | 3.06 | 5.5E-278 |
| SFN | 3.86 | 1.1E-172 | **REG1A** | 3.06 | 3.5E-46 |
| PGC | 3.85 | 1.05E-52 | **ORC1L** | 3.05 | 2.09E-237 |
| CENPF | 3.84 | 1.12E-285 | **HIGD1B** | 3.03 | 1.56E-213 |
| TERT | 3.83 | 3.22E-87 | **NQO1** | 3.01 | 1.56E-194 |
| HJURP | 3.82 | 1.68E-249 | **GNG4** | 3.00 | 3.39E-82 |
| CDKN2A | 3.82 | 1.57E-231 | **B4GALNT1** | 3.00 | 1.47E-157 |
| ASPM | 3.81 | 3.02E-279 | **DCAF4L2** | 3.00 | 1.88E-40 |
| MND1 | 3.79 | 1.25E-221 | **CKAP2L** | 2.99 | 3.47E-215 |
| EXO1 | 3.77 | 3.98E-232 | **OLFML2B** | 2.99 | 6.03E-277 |
| ANLN | 3.76 | 7.84E-255 | **TRAIP** | 2.98 | 1.04E-250 |
| KIFC1 | 3.75 | 1.68E-273 | **CCNE1** | 2.98 | 1.94E-209 |
| PTTG1 | 3.74 | 5.71E-268 | **TNFRSF4** | 2.98 | 2.84E-260 |
| NDC80 | 3.72 | 6.72E-260 | **KIF14** | 2.97 | 7.86E-245 |
| APLN | 3.72 | 1.19E-250 | **COCH** | 2.97 | 2.96E-138 |
| CENPA | 3.72 | 1.38E-203 | **ALG1L** | 2.96 | 3.42E-183 |
| BUB1B | 3.71 | 7.7E-230 | **SHCBP1** | 2.96 | 1.28E-221 |
| ZIC5 | 3.68 | 3.97E-90 | **COX7B2** | 2.94 | 8.56E-44 |
| FOXM1 | 3.68 | 5.85E-282 | **CDCA2** | 2.94 | 1.01E-203 |
| PITX1 | 3.67 | 3.46E-86 | **FAM72D** | 2.94 | 4.51E-208 |
| KIF18B | 3.67 | 2.67E-230 | **BCAN** | 2.92 | 9.37E-123 |
| DEPDC1 | 3.66 | 3.4E-227 | **FAM111B** | 2.91 | 6.39E-265 |
| REG3A | 3.65 | 3.38E-47 | **PLXDC1** | 2.91 | 1.57E-272 |
| XAGE1D | 3.65 | 1.05E-42 | **PSMC3IP** | 2.90 | 4.14E-221 |
| PBK | 3.64 | 8.63E-242 | **EME1** | 2.90 | 1.05E-240 |
| CDK1 | 3.59 | 1.54E-281 | **SIX1** | 2.90 | 2.62E-89 |
| CENPM | 3.58 | 3.82E-238 | **TTC39A** | 2.90 | 2.05E-193 |
| EPR1 | 3.58 | 7.17E-242 | **RECQL4** | 2.88 | 1.56E-299 |
| E2F1 | 3.57 | 3.98E-283 | **PLVAP** | 2.87 | 0 |
| SPC25 | 3.55 | 7.75E-225 | **ELFN2** | 2.87 | 6.76E-132 |
| PLK1 | 3.55 | 1.06E-255 | **CDH13** | 2.87 | 0 |
| MUC13 | 3.53 | 4.41E-147 | **FSTL4** | 2.87 | 7.73E-117 |
| SKA3 | 3.52 | 8.14E-236 | **CA12** | 2.86 | 3.19E-146 |
| HMMR | 3.49 | 9.05E-267 | **MAGEA6** | 2.85 | 9.14E-41 |
| SLC7A11 | 3.49 | 7.6E-162 | **FAM54A** | 2.85 | 1.32E-196 |
| UBE2T | 3.47 | 2.82E-298 | **UBD** | 2.84 | 3.11E-301 |
| CDCA3 | 3.47 | 3.05E-258 | **RDM1** | 2.82 | 3.83E-132 |
| E2F8 | 3.44 | 4.25E-199 | **CEP55** | 2.81 | 1.39E-224 |
| NEIL3 | 3.42 | 2.22E-169 | **CSMD1** | 2.80 | 1.13E-52 |
| KIF18A | 3.42 | 2.77E-202 | **E2F7** | 2.80 | 1.21E-179 |
| PKMYT1 | 3.40 | 5.51E-265 | **C3orf32** | 2.80 | 7.16E-118 |
| TRIP13 | 3.38 | 4.03E-245 | **POLQ** | 2.80 | 1.12E-214 |
| NXPH4 | 3.37 | 8.19E-142 | **MEP1A** | 2.79 | 9.5E-68 |
| CDC6 | 3.34 | 4.36E-278 | **MRAP2** | 2.79 | 1.2E-136 |
| KIF23 | 3.34 | 2.17E-230 | **NCRNA00176** | 2.79 | 5.93E-143 |
| AURKB | 3.34 | 6.55E-246 | **MESP2** | 2.78 | 2.41E-175 |
| MDK | 3.33 | 8.2E-297 | **GNAZ** | 2.76 | 2.12E-212 |
| STC2 | 3.32 | 8.8E-261 |  |  |  |
